# Treatment of Corneal Epithelium Lesions with Plasma Rich in Growth Factors: A Case Series and Implications

**DOI:** 10.3390/healthcare13172184

**Published:** 2025-09-01

**Authors:** Freddy Ortiz, Sofía Cárcamo, Vanessa Souza-Mello, Sergio Echeverría, Cristian Sandoval, José Caamaño

**Affiliations:** 1 Departamento de Especialidades Médicas, Facultad de Medicina, Universidad de la Frontera, Temuco 4811230, Chile; freddyortiz01@gmail.com (F.O.); sergio.echeverria@ufrontera.cl (S.E.); 2 Hospital Dr. Hernán Henríquez Aravena, Temuco 4811230, Chile; 3 Oftoclinic, Clínica Oftalmológica, Temuco 4811230, Chile; 4 Hemovision, Laboratorio de Hemoderivados, Temuco 4811230, Chile; 5 Carrera de Medicina, Facultad de Medicina, Universidad de La Frontera, Temuco 4811230, Chile; s.carcamo03@ufromail.cl; 6 Laboratorio de Inmunohematología y Medicina Transfusional, Unidad de Tecnología Médica, Departamento de Medicina Interna, Facultad de Medicina, Universidad de La Frontera, Temuco 4811230, Chile; cristian.sandoval@ufrontera.cl; 7 Laboratory of Morphometry, Metabolism and Cardiovascular Diseases, Biomedical Center, Institute of Biology, Universidade do Estado do Rio de Janeiro, Rio de Janeiro 22775-000, Brazil; vanessa.souza.mello@uerj.br; 8 Escuela de Tecnología Médica, Facultad de Salud, Universidad Santo Tomás, Los Carreras 753, Osorno 5310431, Chile; 9 Núcleo Científico y Tecnológico en Biorecursos (BIOREN), Universidad de La Frontera, Temuco 4811230, Chile; 10 Carrera de Tecnología Médica, Facultad de Medicina, Universidad de La Frontera, Temuco 4811230, Chile; 11 Centro de Investigación en Medicina de Laboratorio (CeMLab), Facultad de Medicina, Universidad de La Frontera, Temuco 4811230, Chile

**Keywords:** case report, drugs, growth factors, medical therapy, pharmacology

## Abstract

**Purpose:** The human eye is a remarkable organ that develops from many tissues originating from neuroectodermal, ectodermal, and mesodermal sources. If any of these essential ocular tissues are impaired, it can lead to complete vision loss. Thus, the objective of these case studies is to evaluate the impact of growth factor-rich plasma (PRGF) on the healing process of corneal epithelial injuries. **Methods:** This case series includes three patients with corneal epithelium lesions. The patients were treated with hyaluronic acid and matrix-regenerating agent eye drops for seven days. For a minimum of six more weeks, additional cycles of PRGF eye drops were administered. **Results:** All three patients demonstrated improvements in visual acuity, reductions in ocular symptoms, and enhanced ocular surface health. In Patient 1, a good postoperative response was obtained after a second surgery. In Patient 2, after 30 days, the corneal leucoma showed a good response and complete recovery. In Patient 3, the ulcer and visual acuity improved 72 h after the treatment began. **Conclusions:** PRGF eye drops demonstrated efficacy and safety in the treatment of noninfectious corneal ulcers. Additionally, when used in conjunction with other therapies, they have the potential to augment the healing process of corneal ulcers.

## 1. Introduction

The human eye is a complex organ formed through the coordinated development of multiple embryonic tissues, including neuroectodermal, ectodermal, and mesodermal components [1,2]. Disruption in the integrity or function of these essential ocular structures can lead to significant visual impairment or complete vision loss. Among such conditions, corneal disorders, including ulcers, keratitis, and inflammation with or without cellular infiltration, frequently arise due to corneal abrasions or the presence of ocular foreign bodies [2,3]. A corneal ulcer is characterized by an epithelial defect that penetrates the underlying stromal layer. These ulcers are often associated with an inflammatory infiltrate, typically triggered by bacterial invasion through a compromised epithelial barrier [4]. Given the potential for rapid progression and vision-threatening complications, corneal ulceration is considered an ophthalmic emergency [2,5].

A variety of risk factors contribute to the development of corneal infections and ulceration. These include mechanical trauma, prolonged contact lens wear, the use of contaminated eye solutions, and underlying ocular surface abnormalities such as corneal edema secondary to dry eye disease. Systemic factors, such as deficiencies in vitamins A and B12, may further compromise corneal integrity and immune defense mechanisms [6]. The healing process of the cornea involves complex cellular and molecular pathways, which can be influenced by pharmacological agents. Notably, the cornea is densely innervated, and its rich supply of nerve endings plays a critical role in maintaining ocular sensitivity and mediating pain perception [2,7].

Regenerative medicine is a rapidly advancing field offering innovative strategies for the repair or replacement of damaged or lost cells, tissues, and organs resulting from trauma, chronic illness, congenital anomalies, or aging, particularly when endogenous healing mechanisms prove insufficient [2,8,9,10,11]. In ophthalmology, this paradigm shift has spurred increasing interest in engineering-based approaches to restore or regenerate ocular tissues, aiming to combat vision loss associated with degenerative eye diseases, infections, and injuries [12]. Progressive ocular diseases such as cataracts, glaucoma, age-related macular degeneration, and diabetic retinopathy, along with inherited retinal disorders including Stargardt disease, X-linked retinoschisis, retinitis pigmentosa, and Leber congenital amaurosis, remain the leading causes of irreversible blindness. Currently, there are no definitive treatments for many degenerative conditions affecting the retina, cornea, and lens [11,13,14,15,16].

Among the emerging strategies, cell-based therapies, particularly those utilizing stem cells, offer considerable potential to reprogram endogenous cells and promote the regeneration of key ocular structures. This positions cell therapy as a promising intervention for treating diseases involving the cornea, lens, and retina [9,17,18]. In the context of corneal damage, management typically requires a multifaceted approach. Conventional therapies such as lubricating eye drops, antibiotics, contact lens avoidance, or eye patching often fail to resolve the underlying pathology. Additionally, the use of amniotic membranes to repair ulcers or perforations yields a success rate of less than 20% in patients with severe ocular surface disorders [14,19,20].

Researchers have actively sought a substance that closely resembles natural tears, both in composition and function, and has the potential to stimulate the healing of tissues damaged by ocular surface diseases. Plasma-derived products have emerged as highly effective products in managing various ocular surface diseases [14,19,20,21,22] due to plasma containing numerous bioactive proteins, including fibrinogen, and being enriched with growth factors due to the presence of platelets [23,24]. Platelet-rich plasma (PRP), obtained through a two-step centrifugation of autologous blood, concentrates platelets and their associated growth factors, such as thrombospondin, angiogenin, transforming growth factor beta (TGF-β), vascular endothelial growth factor (VEGF), platelet-derived growth factor (PDGF), insulin-like growth factor (IGF), and mitochondrial growth factor, which are essential for initiating cellular regeneration processes [25,26].

Unlike artificial tears, plasma-rich growth factor (PRGF) formulations more closely replicate the pH, osmolarity, and biomechanical characteristics of natural tears [27]. PRGF is characterized by an elevated platelet concentration and contains several growth factors, including PDGF, epidermal growth factor (EGF), IGF-1, TGF-β, basic fibroblast growth factor (bFGF), and VEGF—key mediators of angiogenesis, cell proliferation, and tissue repair [2,28,29]. Platelet-derived products, particularly PRGF, are increasingly applied in the treatment of ocular surface disorders such as dry eye syndrome, recurrent corneal erosions, chemical injuries, limbal stem cell deficiency, corneal ulcers, and chronic epithelial defects [2,30]. Accumulating evidence from preclinical and clinical studies supports the therapeutic potential of PRGF in promoting corneal healing and improving outcomes in a range of ocular surface diseases.

## 2. Case Description and Diagnostic Assessment

### 2.1. Clinical Case Nº1

A 65-year-old male patient, monocular with only the left eye functional, presented with a six-month history of ocular pain, superficial redness, and decreased visual acuity. His medical history was significant for severe ocular trauma in the right eye, resulting in phthisis bulbi. He had no notable comorbidities.

On ophthalmologic examination, the visual acuity of the left eye was reduced to 20/60, with intraocular pressures of 5 mmHg in the right eye and 14 mmHg in the left eye. The biomicroscopy findings were as follows: 1. Right Eye: Signs of atrophy consistent with phthisis bulbi, and 2. Left Eye: Grade 3 nasal pterygium, a clear cornea with mild visual axis involvement, and no other abnormalities.

**Figure 1:**
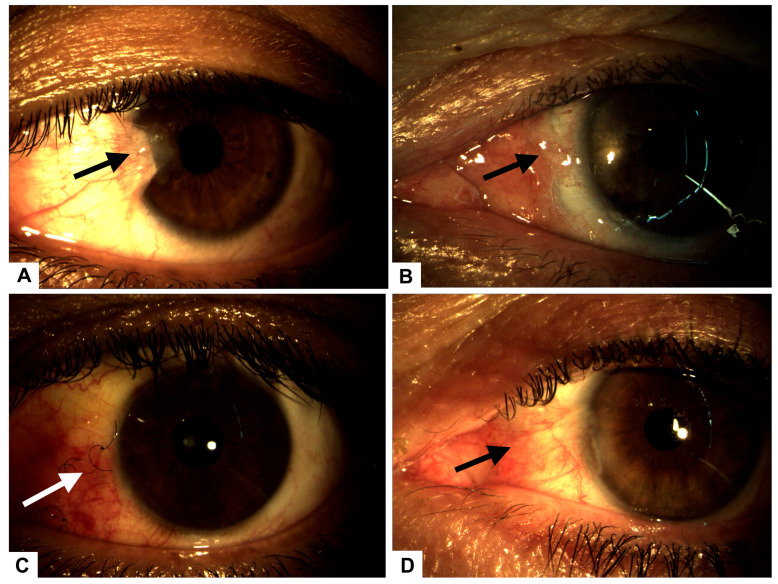
(**A**) Preoperative image showing a Grade 3 nasal pterygium (arrow) in the left eye with mild visual axis involvement. (**B**) Postoperative complication on day 3: graft displacement with complete scleral exposure (arrow), indicative of early-stage scleromalacia. (**C**) Conjunctival surface showing significant recovery and granulation tissue formation (arrow) after 14 days of intensive treatment with autologous serum and PRGF eye drops. (**D**) Complete conjunctival surface restoration (arrow), observed 30 days after initial surgery, following successful placement of a superior conjunctival graft.

Fundoscopy of the left eye revealed an applied retina, a healthy macula, and no lesions. Ocular motility was normal, and there was no monocular diplopia. The refraction results showed neutrality in the right eye and −4.50 CYL at 45° in the left eye, improving the visual acuity to 20/40. The pterygium-induced corneal astigmatism prompted the decision to proceed with surgical intervention (Figure 1A).

The patient underwent pterygium resection surgery in the left eye, employing a classic technique with an autograft secured by five separate stitches. Postoperative care included tobramycin ointment. Follow-up evaluations were conducted on postoperative days 1, 7, and 14. On day 1, the graft and stitches were satisfactorily positioned, while on day 3, complications arose, including graft displacement due to excessive eye movement (Figure 1B), resulting in complete scleral exposure. Intensive treatment with 100% plasma rich in growth factors (PRGF) was initiated, administered as one drop every four hours for 14 days, with bi-daily monitoring (Figure 1C).

By the seventh postoperative day, the graft was poorly positioned with partial loss of three graft sutures. Examination of the anterior segment showed that it remained normal, with no evidence of infection. A decision was made to reposition the graft with additional sutures and a patch, but this intervention was unsuccessful.

On day 10, the patient experienced complete dehiscence of the stitches, loss of the graft, and full scleral exposure. To stimulate granulation tissue formation, autologous serum drops were initiated (1 g every six hours), complemented by a nocturnal humid chamber.

The patient was monitored 48 h after the serum started, and he responded well, showing that granulation tissue began to form. After seven days, the monitoring revealed 100% granulation tissue in the sclera, indicating treatment for secondary scleromalacia.

Fifteen days after initiating serum therapy, the patient showed excellent recovery, with the left eye visual acuity improving to 20/30 and intraocular pressures stabilizing at 5/12 mmHg.

Thirty days post-initial surgery, a superior conjunctival graft was performed, resulting in a favorable postoperative outcome (Figure 1D).

### 2.2. Clinical Case Nº2

A case is presented of a 67-year-old female patient with a known diagnosis of type 2 diabetes mellitus, under metabolic control with 40 units of neutral protamine Hagedorn (NPH) insulin in the morning. Despite treatment, her metabolic control was poor. She also had a history of left peripheral facial paralysis. A fundoscopic examination showed no signs of diabetic retinopathy.

The patient presented with a five-day history of ocular pain, deep redness in the eye, and decreased visual acuity. Visual acuity in the right eye was 20/40, while in the left eye, it had decreased to 20/80. Intraocular pressure was normal on digital tonometry.

**Figure 2:**
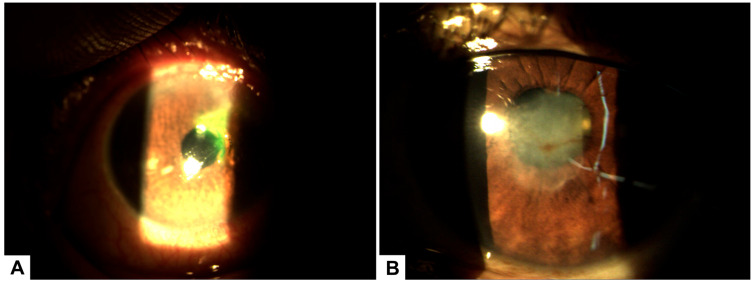
(**A**) Biomicroscopy of the left eye showing a superior corneal ulcer with stromal involvement and associated peripheral pannus prior to initiating treatment with PRGF. (**B**) Biomicroscopy image after 30 days of PRGF therapy, demonstrating complete epithelial healing with residual corneal leucoma and evidence of stromal remodeling.

Biomicroscopy revealed a normal anterior segment in the right eye. In the left eye, however, an upper corneal ulcer with stromal involvement was noted, along with a peripheral pannus adjacent to the ulcer. No anterior chamber reaction or hypopyon was present (Figure 2A).

Corneal optical coherence tomography (OCT) confirmed stromal involvement without evidence of corneal perforation, consistent with exposure keratopathy secondary to peripheral facial paralysis. Corneal thickness was reduced by approximately two-thirds.

Initial management included ocular surface hydration using preservative-free 0.4% sodium hyaluronate drops every six hours, along with gel-based lubricants for 7 days. However, no significant clinical improvement was observed, and the corneal ulcer persisted. As there were no clinical signs of infection, antibiotic therapy was not initiated.

**Figure 3:**
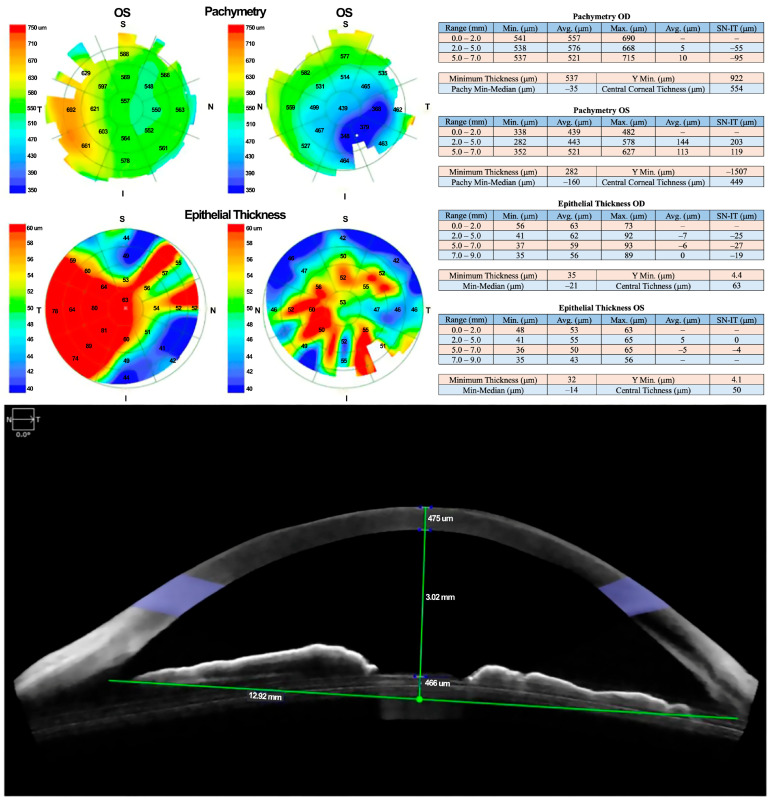
Follow-up OCT revealed early signs of stromal thickening, indicating a regenerative response to PRGF therapy.

Due to a lack of adequate response, treatment with 100% PRGF eye drops was initiated at a dose of one drop every four hours for 14 days. Corneal pachymetry and OCT performed 48 h after initiating PRGF showed early signs of response, including stromal thickening indicative of regenerative activity (Figure 3).

Seventy-two hours after the start of PRGF therapy, the patient demonstrated further clinical improvement, with the development of a corneal leucoma over the previously ulcerated area. After 7 days of treatment with PRGF, visual acuity in the affected eye improved to 20/40. Complete epithelial healing occurred within 14 days, although a residual corneal leucoma caused some visual disturbance.

To promote epithelial remodeling and reduce the leucoma, topical fluorometholone 0.1% was initiated. After 30 days of therapy, the leucoma showed significant improvement, and the corneal surface achieved complete recovery (Figure 2B).

### 2.3. Clinical Case Nº3

**Figure 4:**
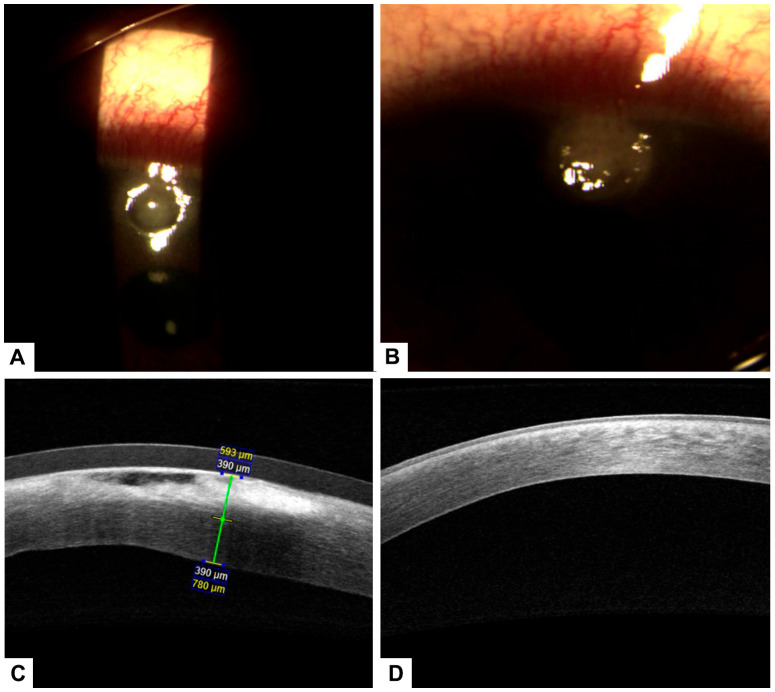
(**A**) Pterygium prior to treatment. (**B**) Conjunctival surface recovery two days after treatment, with early epithelial healing observed. (**C**) Corneal surface showing a stromal ulcer before initiation of treatment with PRGF. (**D**) Significant improvement of the corneal surface after PRGF therapy, with epithelial closure and reduction of inflammation.

A case of a 55-year-old male patient with a prior diagnosis of type 2 diabetes and poor metabolic control who uses NPH insulin and crystalline insulin. He does not report regular fundus examinations. He reports a seven-day foreign body sensation in the left eye, associated with ocular pain, deep red eyes, purulent discharge, and decreased visual acuity. The visual acuity of the right eye (20/30) decreased, as did the visual acuity of the left eye (20/60). The right eye had an intraocular pressure of 12, while the left eye did not have any. During the ophthalmologic examination, we extracted a corneal foreign body from the corneal stroma with difficulty and noted a corneal ulcer with stromal involvement; however, the symptoms remain. A fundus examination was performed, showing mild non-proliferative diabetic retinopathy without macular involvement. The decision was made to initiate treatment with antibiotics and 4% hyaluronic acid. However, after 72 h of control, there was no significant recovery, and the traumatic corneal ulcer persisted. In the new examination, the corneal stroma was involved in a corneal ulcer. Consequently, the decision was made to continue with 100% PRGF every 4 h, complemented by topical antibiotic treatment, which showed significant improvement. This treatment was carried out for 14 days topically. Changes were evident on the second day after starting plasma therapy (Figure 4). The ulcer and visual acuity improved 72 h after the treatment began, and the left eye’s visual acuity remained unchanged at 20/30.

## 3. Treatment

The only thing that helped the patients was hyaluronic acid (0.4%) and matrix regenerating agent (MRA) eye drops [Cacicol20^®^, OTR3, Paris, France] four times a day for seven days. It was thought to be possible to combine topical medication with Tobramycin antibiotic therapy. At the onset of treatment, the use of corticosteroids to enhance re-epithelialization was not taken into consideration. However, despite topical application four times a day for six weeks (one cycle lasting six weeks), no reduction in the dry eye symptoms or closure of the corneal ulcer was observed. The administration of additional cycles of PRGF eye drops was contingent upon the degree of closure of the corneal ulcer. When the corneal ulcer had closed, the MRA array was stopped; nevertheless, PRGF eye drops had to be used for at least six more weeks in order to stabilize the dry eye disease symptoms. When the medical doctor thought it was necessary, additional topical therapies (corticosteroids or artificial tears) were administered to the patients. To assess the safety of these treatments, all untoward incidents or problems that arose during the trial were documented. No inconveniences were reported by the patients.

## 4. PRGF Preparation

After the patient provided informed consent, blood was drawn and placed in acid citrate dextrose (ACD-A) in 8.5 mL tubes. The samples underwent a 15 min, 300× *g* centrifugation at ambient temperature. It was possible to avoid the buffy coat after centrifugation, and a micropipette was used to collect the whole column of growth factor-rich plasma in a laminar flow gate. After activating the entire PRGF volume with calcium chloride, it was incubated for one hour at 37 °C. Following a 10 min centrifugation at 1000 g, the growth factor-enriched supernatants were gathered by aspiration. They were then filtered through a 0.2 mm filter pore size (Thermo Fisher Scientific, Waltham, MA, USA), dispensed into a multidose eye dropper (Novelia^®^, Nemera Development S.A., Lyon, France) without the need for preservatives, and stored at 4 °C.

## 5. Follow-Up

**Figure 5:**
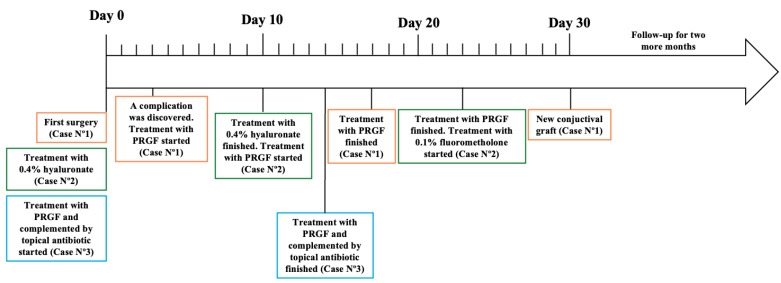
Follow-up of each case presented in this study.

Initially, after being monitored weekly for the first month, the participants were then seen every two weeks until re-epithelialization was achieved at both the conjunctiva and cornea, depending on the clinical case. The patients were followed until defect closure and control of dry eye symptoms were achieved. This follow-up was monthly for 3 months, and during this follow-up period, intervention adherence and tolerability data were collected from medical records, photographs, and questionnaires. No adverse effects were reported. A summary of the follow-up can be observed in Figure 5 and Table 1.

## 6. Discussion

### 6.1. Corneal Ulcer Management and the Role of PRGF

Corneal ulceration refers to the breakdown of the corneal epithelium, often resulting from infections but also associated with trauma, ocular dryness, prolonged use of scleral lenses, and inflammatory or allergic ocular conditions [31,32]. The primary goals in managing corneal ulcers are to reduce the risk of infection, promote tissue regeneration, and prevent cicatricial changes that may compromise visual acuity. However, the diagnosis and treatment of noninfectious corneal ulcers remain challenging, as conventional therapies, such as ocular lubricants, topical immunomodulators, and ointments, are often insufficient [24,33].

In the context of ocular surface disorders, neurotrophic keratopathy or neurotrophic ulcers provide a significant issue. These arise from trigeminal nerve dysfunction and are marked by diminished corneal sensitivity and compromised healing, frequently leading to persistent epithelial abnormalities and stromal ulcers [34]. Case Nº2 involved a patient with peripheral facial paralysis, a condition typically linked to diminished corneal sensibility, rendering it a probable instance of a neurotrophic ulcer. The favorable reaction to PRGF underscores its promise as a restorative alternative in neurotrophic keratopathy by reinstating epithelial integrity and promoting healing due to its abundant growth factor content [35].

Wound healing on the ocular surface is a complex process in which platelets play a critical role by releasing growth factors that attract fibroblasts to the injury site and stimulate tissue regeneration. In vitro studies have demonstrated that platelet-rich plasma (PRP) enhances the biological activity of ocular fibroblasts [2,36,37]. Nevertheless, myofibroblasts, while essential in tissue repair through extracellular matrix production and scar formation, can contribute to long-term fibrotic changes and corneal opacity if their activity persists during healing [38,39].

Recent studies have shown that blood-derived therapies not only inhibit excessive myofibroblast activation but also promote the migration and proliferation of corneal and conjunctival fibroblasts. This dual action facilitates regeneration while minimizing scarring [40]. Among these therapies, autologous fibrin membranes enriched with growth factors have demonstrated efficacy in treating severe or refractory corneal ulcers, particularly those unresponsive to standard treatment modalities. PRGF, used alone or in combination with collagen or amniotic membranes, has been successfully applied to promote epithelial closure and corneal healing [38,39,40,41,42].

Clinical evidence supports the therapeutic benefit of PRGF eye drops, with one study reporting a 92% improvement in inflammation and ocular discomfort among patients with refractory corneal ulcers treated with six daily applications [39,40]. Unlike autologous serum (AS) drops, PRGF is devoid of erythrocytes and leukocytes, thereby reducing exposure to proinflammatory cytokines and offering a safer alternative, particularly in patients with autoimmune conditions [35,43].

PRGF is rich in bioactive compounds such as PDGF, EGF, vitamin A, fibronectin, antimicrobial agents, and anti-inflammatory molecules—all of which contribute to enhanced cell migration, differentiation, and tissue repair [26,44,45,46]. Additionally, PRGF is prepared under sterile, controlled conditions, ensuring its stability for up to six months and reducing dependency on external blood banks or compounding pharmacies [47,48,49].

### 6.2. Clinical Applications and Considerations

Prophylactic antibiotics, corticosteroids, and artificial tears are commonly employed in the management of noninfectious corneal ulcers. Although these treatments are generally effective, they can be costly and may lead to tolerance-related issues. In contrast, PRGF eye drops have demonstrated excellent safety and efficacy when used as an adjunctive therapy for noninfectious corneal ulcers, significantly promoting corneal healing.

However, PRGF therapy should be discontinued if serious adverse events, such as severe allergic reactions or microbiological contamination, occur. The use of PRGF is contraindicated in patients with the following conditions: (a) active bacterial infections, (b) hepatitis B infection (unless the patient is immune and HBsAg-negative), (c) positive serological markers for HIV, HTLV I/II, or hepatitis C virus (HCV), and (d) severe cardiovascular disorders.

Although artificial tears and matrix-regenerating agents such as Cacicol20^®^ (Laboratoires Théa, Clermont-Ferrand, France) are commonly employed for corneal epithelial lesions, they frequently lack the multifaceted regenerative capabilities present in autologous products like PRGF. This study indicates that not all patients had previously responded to hyaluronic acid and matrix-repairing drops prior to initiating PRGF, highlighting its greater efficacy in refractory or non-responsive ulcers [22]. However, comparison trials are absent, and forthcoming studies should assess PRGF directly against commercial alternatives in randomized controlled environments to validate these results [44].

### 6.3. Limitations

As a descriptive and observational design, this clinical case series presents inherent limitations. First, epidemiological metrics such as incidence, prevalence, or risk ratios cannot be derived due to the absence of a representative population sample and control group. Consequently, these findings are not generalizable, and any hypothesis generated must be confirmed by more robust study designs. Furthermore, as each case report is retrospective, this may lead to incomplete clinical data and susceptibility to information bias.

## 7. Conclusions

PRGF represents a promising therapeutic strategy for noninfectious corneal ulcers, offering advantages such as improved tissue healing, reduced scarring, and minimal proinflammatory risks. Its preparation in-office further enhances accessibility, providing an effective and patient-friendly alternative to traditional therapies.

This case series highlights the innovative application of PRGF in real-world, intricate instances, including neurotrophic ulcers and post-surgical sequelae including scleromalacia. The demonstration of initial granulation tissue formation, complete corneal healing within two weeks in diabetic individuals, and re-epithelialization despite graft failure underscores the regenerative benefits of PRGF, particularly when conventional therapies have proven ineffective. The practical applications of PRGF therapy enhance the existing comprehension and utilization in ophthalmic regenerative medicine [30,41].

## Figures and Tables

**Table 1:** Summary of the included case series.

Parameter	Case Nº1	Case Nº2	Case Nº3
Age/Sex	65/male	67/female	55/male
Relevant history	Monocular (right eye phthisis bulbi from trauma)	Type 2 diabetes, left peripheral facial paralysis	Type 2 diabetes, poor control, no regular fundus exam
Initial symptoms	6-month ocular pain, redness, decreased visual acuity in left eye	Five-day ocular pain, deep red eyes, low visual acuity (left eye)	Seven-day foreign body sensation, pain, discharge, decreased vision (left eye)
Visual acuity (initial)	Right: NA (phthisis)/left: 20/60 (corrected to 20/40)	Right: 20/40 → 20/20/left: 20/80	Right: 20/30/left: 20/60
Biomicroscopy	LE: nasal pterygium grade 3, clear cornea with mild involvement	LE: upper corneal ulcer, stromal involvement, pannus, no chamber reaction	LE: corneal ulcer with stromal involvement, foreign body removal attempted
OCT findings	Not performed	LE: stromal ulcer without perforation, 2/3 thickness loss	LE: stromal involvement with persistent ulcer
Treatment (initial)	Pterygium resection with autograft, tobramycin ointment	Ocular moisturizers (hyaluronate 0.4%) for 7 days	Antibiotics and 4% hyaluronic acid
Complications/failure	Graft displacement → scleral exposure → unsuccessful repositioning	No response to initial hydration therapy	No response after 72 h of antibiotic/hyaluronic acid
PRGF therapy	Initiated post-graft failure (1 drop q4h for 14 days), followed by autologous serum, then graft	PRGF 1 drop q4h for 14 days → epithelial healing with residual leucoma → steroid remodeling therapy	PRGF 1 drop q4h + antibiotics for 14 days
Follow-up findings	Granulation after 48 h serum, full recovery by 30 days	Response by 48–72 h, full healing in 14 days, corneal remodeling with fluorometholone	Ulcer improvement by day 2, VA improved to 20/30 by day 3
Final visual acuity	Left eye: 20/30	Left eye: 20/40	Left eye: 20/30
Outcome	Favorable graft recovery, stable IOP, and visual acuity	Leucoma formed but remodeling successful, complete recovery	Significant recovery with PRGF, no further complications

## Data Availability

The original contributions presented in this study are included in the article. Further inquiries can be directed to the corresponding author.
